# Genetic Homogeneity of Measles Viruses Associated with a Measles Outbreak, São Paulo, Brazil, 1997

**DOI:** 10.3201/eid0808.020040

**Published:** 2002-08

**Authors:** Maria I. Oliveira, Paul A. Rota, Suely P. Curti, Cristina A. Figueiredo, Ana M. S. Afonso, Marcia Theobaldo, Luiza T.M. Souza, Stephanie L. Liffick, William J. Bellini, Jose C. Moraes, Klaus E. Stevien, Edison L. Durigon

**Affiliations:** * Adolfo Lutz Institute, São Paulo; Brazil; † Centers for Disease Control and Prevention, Atlanta, Georgia, USA; ‡ Center for Epidemiological Surveillance, São Paulo, Brazil; § Institute of Biomedical Science, São Paulo, Brazil

During a resurgence of measles in São Paulo, Brazil, in 1997, >40,000 cases (peak incidence rate of 246/100,000 inhabitants) and 42 measles-related deaths were reported. Reverse transcriptase-polymerase chain reaction and nucleotide sequencing were used to analyze specimens from patients who had typical clinical measles infection during this outbreak and from six patients who had had measles in 1995 and 1996. Although wild-type measles viruses (genotypes D5 and D6) were present in São Paulo before this resurgence, we detected only D6 viruses. The genotype D6 viruses isolated during this outbreak had identical sequences to genotype D6 viruses isolated in other parts of Brazil and South America in 1997 and 1998, suggesting that a single chain of transmission was responsible. We also identified genotype A viruses in two vaccine-associated cases from 1995 and 1996. Our findings extend the knowledge of the circulation patterns of measles virus in South America, contributing to measles control efforts in the Americas.

The Pan American Health Organization (PAHO) reported record low numbers of measles cases for 2000–2001, reflecting the success of measles control programs in the Western Hemisphere (1). In the most populous country in the region, the United States, indigenous transmission of measles virus has been eliminated since 1993, and only 3 of 41 countries in the region reported indigenous measles transmission during 2001 (1,2). Despite this success, measles remains an endemic disease in many areas of the world. The World Health Organization (WHO) estimates that approximately 45 million cases of measles and >800,000 measles-related deaths continue to occur annually (3,4). Sporadic measles outbreaks still occur in both developed and undeveloped countries that have failed to maintain adequate immunization levels. These outbreaks, such as the one that occurred in São Paulo, Brazil, in 1997, provide an opportunity to study the virologic and epidemiologic aspects of measles transmission in vaccinated populations.

Before the introduction of vaccine, measles was a substantial public health problem in Brazil, representing a major cause of death among young children. Even after the introduction of vaccine, measles epidemics continued to occur in Brazil. For example, the epidemics of 1984 and 1986 had incidence rates of 63/100,000 and 97/100,000, respectively. PAHO launched an aggressive measles control program for the Americas in 1991 that included one-time national catch-up campaigns for all children, routine “keep-up” vaccination of infants at 1 year of age, and follow-up campaigns at 3- to 5-year intervals. In Brazil, this strategy resulted in a 93% reduction in the incidence of measles in Brazil from 1991 through 1996. For the 4-year period 1993–1996, <1,000 cases were reported annually. However, during the resurgence of measles in 1997 in São Paulo, 42,055 confirmed cases of measles (23,907 laboratory confirmed), were reported, with a peak incidence rate of 246/100,000 inhabitants. Most of the cases were in adults living in metropolitan São Paulo. Forty-two deaths caused by measles were reported during this epidemic (5–7).

Virologic surveillance can help to identify the transmission pathways of measles virus and is an important component of measles surveillance activities (8–10). Sequence analysis of measles viruses has shown that distinct lineages of wild-type viruses exist and co-circulate, and WHO currently recognizes 20 measles genotypes (11–13). The epidemiologic aspects of the measles outbreak in São Paulo have been described (5–7). The purpose of this report is to present the genetic characteristics of the measles viruses responsible for the resurgence of measles in São Paulo, Brazil, during 1997. This outbreak provided an opportunity to study the transmission of viruses in an area that had previously had good measles control but then experienced a rapid resurgence in cases. Our study showed that a single genotype of virus, D6, was responsible for the outbreak and that viruses from this chain of transmission subsequently spread to other countries in South America.

## Materials and Methods

### Specimens

Samples from patients with laboratory-confirmed measles were analyzed (Table). Six of the samples were serum specimens; the rest were heparinized, whole-blood specimens. Peripheral blood mononuclear cells (PBMC) were purified from whole blood by using Ficoll-Hypaque gradient centrifugation. The PBMC were washed three times with phosphate-buffered saline before being resuspended in Dulbecco’s minimal essential medium (DMEM) containing 10% fetal calf serum (FCS). Aliquots of PBMC were cryopreserved by using standard procedures (14).

**Table:** Summary of 41 measles specimens analyzed, São Paulo, Brazil, March 1995–October 1997.

Name^a^	ID^b^	Age	Date of specimen collection	Vaccination status^c^	Genotype^a^
MVs/São Paulo.BRA/10.95	205*	9 m	03/09/95	02/24/95	A
MVs/São Paulo.BRA/35.95/2	258*	9 m	08/30/95	Yes	D5
MVs/São Paulo.BRA/35.95/1	2793*	9 m	08/29/95	08/19/95	D6
MVs/São Paulo.BRA/3.96	141*	9 m	01/15/96	01/03/96	A
MVs/São Paulo.BRA/5.96	431*	9 m	02/26/96	02/08/96	D6
MVs/São Paulo.BRA/27.96	1490*	9 m	07/03/96	06/15/96	D6
MVs/São Paulo.BRA/4.27	106	25 yr	01/20/97	No	D6
MVs/São Paulo.BRA/6.97	267	20 yr	02/04/97	Yes	D6
MVs/São Paulo.BRA/9.97	503	30 yr	03/01/97	Unknown	D6
MVs/São Paulo.BRA/10.97	561	24 yr	03/06/97	Unknown	D6
MVs/São Paulo.BRA/11.97	661	20 yr	03/11/97	No	D6
MVs/São Paulo.BRA/12.97/3	760	27 yr	03/18/97	No	D6
MVs/São Paulo.BRA/12.97/1	764	23 yr	03/17/97	Unknown	D6
MVs/São Paulo.BRA/12.97/2	765	31 yr	03/17/97	No	D6
MVi/São Paulo.BRA/12.97/4	802	29 yr	03/18/97	No	D6
MVs/São Paulo.BRA/12.97/5	862	26 yr	03/18/97	Unknown	D6
MVs/São Paulo.BRA/14.97/2	1045	6 m	04/02/97	No	D6
MVs/São Paulo.BRA/14.97/3	1048	27 yr	04/02/97	No	D6
MVs/São Paulo.BRA/14.97/1	1084	3 m	04/01/97	No	D6
MVs/São Paulo.BRA/15.97/1	1225	4 m	04/07/97	No	D6
MVi/São Paulo.BRA/14.97/4	1139	22 yr	04/03/97	No	D6
MVs/São Paulo.BRA/15.97/2	1202	2 yr	04/10/97	Unknown	D6
MVs/São Paulo.BRA/17.97/1	1398	15 yr	04/23/97	No	D6
MVi/São Paulo.BRA/17.97/3	1494	33 yr	04/27/97	No	D6
MVs/São Paulo.BRA/17.97/2	8954	3 yr	04/25/97	Yes	D6
MVs/São Paulo.BRA/32.97	9462	20 yr	08/21/97	Unknown	D6
MVi/São Paulo.BRA/33.97/1	9463	25 yr	08/12/97	No	D6
MVs/São Paulo.BRA/33.97/2	9778	24 yr	08/13/97	Unknown	D6
MVs/São Paulo.BRA/30.97	9786	-	07/22/97	Yes	D6
MVs/São Paulo.BRA/34.97/4	10085	3 m	08/21/97	No	D6
MVs/São Paulo.BRA/33.97/5	10171	30 yr	08/15/97	No	D6
MVs/São Paulo.BRA/33.97/6	10172	26 yr	08/15/97	Unknown	D6
MVs/São Paulo.BRA/33.97/3	10173	5 m	08/14/97	No	D6
MVs/São Paulo.BRA/33.97/4	10175	24 yr	08/14/97	Unknown	D6
MVs/São Paulo.BRA/33.97/8	10179	28 yr	08/16/97	Unknown	D6
MVs/São Paulo.BRA/33.97/9	10181	22 yr	08/17/97	Yes	D6
MVs/São Paulo.BRA/34.97/1	10242	25 yr	08/19/97	No	D6
MVs/São Paulo.BRA/33.97/7	10660	20 yr	08/15/97	Yes	D6
MVs/São Paulo.BRA/34.97/2	10661	3 yr	08/19/97	No	D6
MVs/São Paulo.BRA/34.97/3	10705	31 yr	08/21/97	Unknown	D6
MVi/São Paulo.BRA/42.97	35175	4 m	10/14/97	No	D6

^a^Sequence name and genotype designation based on nomenclature established by World Health Organization (11). ^b^ID, specimen identification number. All samples were from purified peripheral blood mononuclear lymphocytes except for serum samples, designated by an asterisk. ^c^Date of last measles vaccination, if known.

### Virus Isolation

B95a cells (15) were injected with 0.2 mL of the clinical samples and maintained in DMEM, 0.2 mM L-glutamine/100 mL and gentamicin 40 mg/mL, in 2% FCS at 37°C. Samples with no apparent cytopathic effect (CPE) after 3 passages were discarded as negative for virus isolation. If CPE was observed, the cells were harvested by centrifugation when CPE was maximal, and RNA was extracted from the cell pellet. Five measles viruses were isolated from PBMC collected during the epidemic.

### RNA Extraction, Polymerase Chain Reaction, and Sequencing

RNA was extracted from serum, PBMC, or infected cell pellets by the guanidinium acid-phenol method (16). cDNA corresponding to the nucleotides coding for the carboxyl terminus of the nucleoprotein (N) or the full-length open reading frame for the hemagglutinin (H) gene were synthesized by using avian myeloblastosis virus reverse transcriptase (RT) and amplified by polymerase chain reaction (PCR), and the sequences of the PCR products were obtained as described previously (17,18). Sequence data were analyzed by using version 10.1 of the Genetics Computer Group Sequence Analysis Software Package (Accelrys Inc., Madison, WI) (18) and phylogenetic analyses were performed with PAUP version 4.0 (19). A reference N gene sequence was deposited in GenBank under accession number AF495863.

## Results

**Figure 1:**
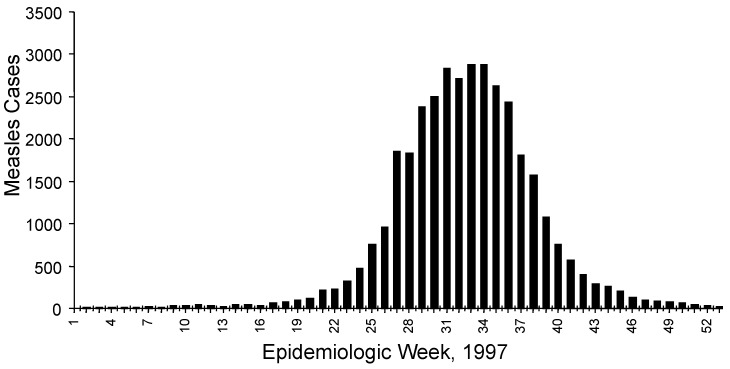
Number of measles cases, by week, São Paulo, Brazil, 1997.

We obtained genetic information from specimens that were collected in São Paulo from March 1995 to October 1997 (Table). During 1995 and 1996, relatively few measles cases were reported in São Paulo; six acute-phase serum samples were obtained from confirmed, sporadic cases that occurred as early as 2 years before the outbreak. In 1997, a resurgence of measles occurred in São Paulo, beginning in the fall and peaking in late winter (Figure 1). Twenty-three samples were obtained from cases that occurred during the first 42 weeks of 1997; 11 of these samples were obtained in August 1997 during the peak of the outbreak (Table). We obtained most of the outbreak specimens from young adults, which is consistent with the observation that most of the measles cases occurred in unvaccinated, young adults (6). Measles-specific immunoglobulin (Ig) M responses were detected in all the patients listed in the Table, except one. The serum sample from case number 802 was negative, probably because it was obtained <3 days after rash onset. However, this case was confirmed by a positive virus isolation (Table).

**Figure 2:**
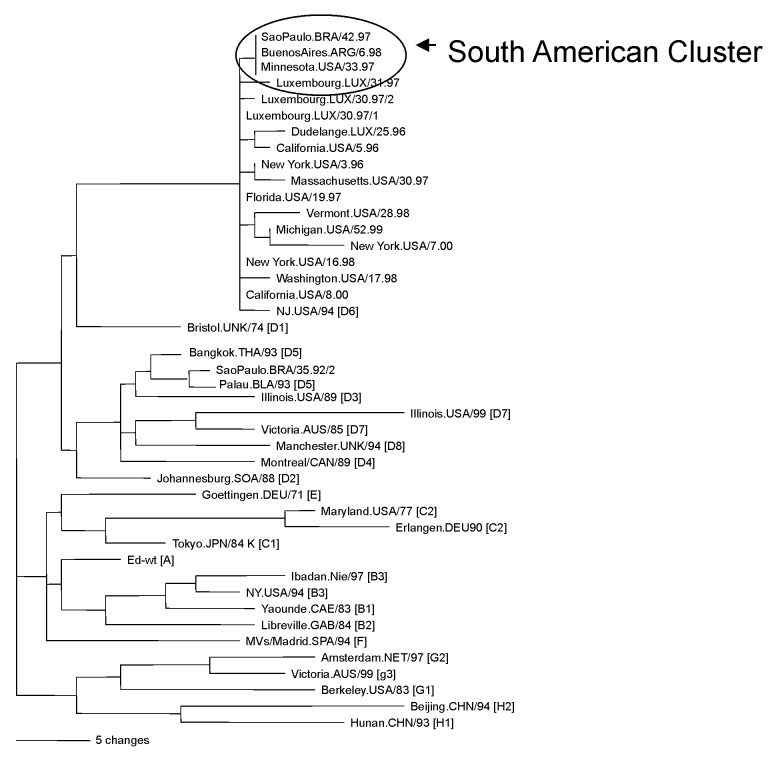
Phylogenetic relationships of the N gene sequences from genotype D6 viruses from Brazilian, South American, and European sources. Tree shows representative genotype D6 sequences from Brazil, Argentina, Uruguay, and Bolivia, and viruses imported into the United States from Brazil (South American cluster) compared with genotype D6 sequences of viruses isolated in Europe or imported into the United States from European sources. Also shown are the sequences of the World Health Organization reference strains for each genotype.

Virus isolation was not attempted for the serum samples, but five virus isolates were obtained from the PBMC specimens (Table) obtained during 1997. The sequences coding for the carboxyl terminus of the nucleoprotein (456 nucleotides) of these isolates were compared with the sequences of the WHO reference strains (12). The results indicated that these Brazilian viruses all had identical N gene sequences and were members of genotype D6 (Figure 2). The N gene sequence of MVi/SãoPaulo.BRA/42.97/35175 has been deposited in GenBank (accession no. AF495863) as a reference sequence for this outbreak. The complete sequence of the H gene was also obtained for MVi/SãoPaulo.BRA/42.97/35175. Comparison of this H gene sequence with the sequences of the H genes from the WHO reference strains confirmed the placement of these viruses in genotype D6 (data not shown).

For the rest of the specimens from 1997, nucleotide sequences were obtained from PCR products that were amplified directly from RNA extracted from the PBMC specimens. In most cases, we had to perform a nested PCR reaction to get sufficient material for sequencing. All specimens obtained in 1997, either just before or during the peak of the outbreak, were placed in genotype D6. Most of the N gene sequences obtained from these specimens were identical to each other and to the N gene sequences obtained from the five isolates from 1997. Only three of the specimens had sequences that differed from the majority sequence by a single nucleotide.

We were also able to amplify measles-specific PCR products from the serum samples obtained from sporadic measles cases that occurred in 1995 and 1996. Two of these specimens had nucleotide sequences in genotype A, which contains all vaccine viruses. Both specimens were obtained from 9-month-old infants who had received their measles vaccination 12–13 days before collection of the serum specimen. One of the samples obtained in 1995 had an N gene sequence in genotype D5, while the other three serum samples had sequences in genotype D6 identical to the D6 sequences obtained during the outbreak. These data showed that D6 viruses and wild-type viruses from genotypes other than D6 were present in São Paulo as early as 2 years before the peak of the outbreak in 1997.

We compared the N gene sequences from the genotype D6 viruses in the São Paulo cases with N gene sequences from genotype D6 viruses isolated in other parts of South America (20–24) and the world (Figure 2). For simplicity, three representative sequences from the South American viruses are shown in the phylogenetic tree. The sequences from the São Paulo outbreak were identical to those obtained from viruses isolated in Brazil (i.e.,Valenca, Rio de Janeiro, Belo Horizonte, Angra do Reis, and Curitiba) (20,21), Argentina (22,23), Uruguay (24), and Bolivia in 1997 and 1998. This homogenous group also included two viruses that were imported into the United States from Brazil in 1997 (25). The sequences from genotype D6 viruses imported into the United States (26, unpub. data) from European sources and genotype D6 viruses isolated in Europe (27,28) were slightly different from the South American D6 group. We found that the sequences from the South American viruses were most closely related to a genotype D6 virus isolated in Luxembourg in 1997, although no foreign source was identified for the outbreak in Brazil. Overall, the N genes sequences of D6 viruses within the South American group differed by no more than 0.4% but differed from other genotype D6 sequences by as much as 1.4%.

## Discussion

This report presents the virologic surveillance data from the measles outbreak that occurred in São Paulo, Brazil, during 1997. Sequence data from the specimens obtained during the outbreak indicated the presence of measles viruses belonging to genotype D6, which is one of the prevalent genotypes of measles virus in Europe. Frequently associated with imported measles cases from European countries (8,26), D6 viruses have been isolated in the United Kingdom, Spain, Germany, Russia, Poland, Denmark, and Luxembourg (27–31). During 1997–2000, these genotype D6 viruses were imported into the United States from Italy, Greece, Ukraine, Croatia, Cyprus, and the United Kingdom, and from the large measles outbreak that occurred in São Paulo (Rota, unpub. data). However, because neither conventional case nor virologic surveillance was conducted in Brazil before the start of the measles control program in 1991, it is not possible to determine if the D6 viruses were recently introduced into Brazil by importation or if they represent continued circulation of viruses indigenous to Brazil. Our data show that D6 viruses indistinguishable from the D6 viruses isolated during the São Paulo outbreak were responsible for sporadic measles cases in São Paulo for at least 2 years before the outbreak. In a large urban area such as São Paulo, the virus will likely be continually present either from low-level circulation or frequent importation. In late 1997, a change occurred in the epidemiologic circumstances, resulting in a large outbreak of the measles virus. Factors that contributed to the outbreak included the failure to conduct a timely follow-up vaccination campaign in 1995 and the low level of vaccination coverage among infants achieved by routine health services. Additionally, a large number of unvaccinated young adults who had escaped measles infection had moved to São Paulo from rural areas. The high population density in São Paulo also facilitated rapid spread of the virus (7).

Laboratory-based surveillance for measles was established in Brazil in the early 1990s. Although virologic surveillance was certainly incomplete, some information is available about measles genotypes present in Brazil before the 1997 outbreak in São Paulo. Genotype D5 was detected in a sporadic case in São Paulo in 1995 and in sporadic cases that occurred in the state of Bahia in 1996 (21). Genotype D5 viruses that circulate in Japan have been associated with importation of viruses from Japan into other countries (26,32,33). The source of the genotype D5 viruses detected in São Paulo and Bahia was not identified. Genotype C2 viruses were detected in a small outbreak in the state of Santa Catarina in 1996 (21). C2, another indigenous genotype in Europe, is often associated with imported cases in other parts of the world (27–31).

Fever and rash occur in approximately 5% of measles vaccine recipients (34). In areas where wild-type virus is circulating, vaccine reactions may be classified as measles cases. Distinguishing a vaccine reaction from a measles case is not possible with the currently available serologic methods. Only the genetic analysis of virus isolates can confirm the presence of either a vaccine or wild-type virus. In this study, two vaccine viruses were detected in persons who had received vaccine 12 and 13 days before collection of specimens. In vaccine reactions, rash and fever typically occur 7 to 12 days after vaccination; the virus can be detected in circulating lymphocytes even after the rash and fever have resolved.

The situation in Brazil is now very similar to that in the United States in the early 1990s. From 1989 to 1991, the United States experienced a resurgence of measles, with >50,000 reported cases. Virologic surveillance suggested that virus from a single genotype, D3, had seeded the entire country. As was the case with the D6 viruses from South America, the D3 viruses from many different areas in the United States had nearly identical sequences (8). Therefore, we conclude that when measles outbreaks occur in areas that have good measles control, genetic analysis of viruses will confirm the presence of a single chain of transmission. In contrast, in areas where measles control programs are less advanced, genetic studies have identified multiple chains of transmission within a genotype (17).

Initiation of a two-dose vaccination schedule, along with the success of the PAHO strategy in the Americas, resulted in historically low numbers of cases in the United States in the years following the resurgence there; both standard epidemiologic surveillance and molecular epidemiologic surveillance have documented the sustained interruption of transmission of the genotype D3 viruses (1,2,8,35–37). Similarly, the pattern of viral genotypes found in Brazil and other parts of South and Central America will help to assess the success of the PAHO measles control programs. Genotype D6 viruses were circulating in Haiti and the Dominican Republic during 2001 but have not been isolated from ongoing chains of transmission in any other countries in the region (36). During 2002, virologic surveillance showed that measles cases imported into El Salvador from Europe were genotype D7 (38). As the number of measles cases continues to decrease in the Americas, increasing virologic surveillance activities will be important. This virologic surveillance will document the interruption of indigenous transmission of the genotype D6 viruses in Brazil and in other parts of South America.

## References

[1] Centers for Disease Control and Prevention. Progress toward interrupting indigenous measles transmission - region of the Americas, January–November 2001. JAMA. 2002;287:709–10. 10.1001/jama.287.6.709 11855385

[2] Centers for Disease Control and Prevention. Measles—United States. MMWR Morb Mortal Wkly Rep. 2000;49:557–60. 10921493

[3] World Health Organization. Expanded Programme on Immunization. Measles control. Geneva: The Organization; document WHO/EPI/GEN/ 92.3; 1992. p. 1–34.

[4] de Quadros CA, Olive JM, Hersh BS, Strassburg MA, Henderson DA, Brandling-Bennett D, Measles elimination in the Americas. Evolving strategies. JAMA. 1996;275:224–9. 10.1001/jama.275.3.224 8604176

[5] Centers for Disease Control and Prevention. Progress toward elimination of measles from the Americas. MMWR Morb Mortal Wkly Rep. 1998;47:189–93. 9531021

[6] Camargo MCC, de Moraes JC, Souza VA, Matos MR, Pannuti CS. Predictors related to the occurence of a measles epidemic in the city of São Paulo in 1997. Rev Panam Salud Publica. 2000;7:359–65. 10.1590/S1020-49892000000600001 10949895

[7] Hersh BS, Tambini G, Nogueira AC, Carrasco P, de Quadros CA. Review of measles surveillance data in the Americas, 1996–1999. Lancet. 2000;355:1943–8. 10.1016/S0140-6736(00)02325-4 10859039

[8] Rota JS, Health JL, Rota PA, King GE, Jin L, Brown DWB, Molecular epidemiology of measles virus: identification of pathways of transmission and implications for measles elimination. J Infect Dis. 1996;73:32–7. 10.1093/infdis/173.1.32 8537679

[9] Bellini WJ, Rota PA. Genetic diversity of wild-type measles viruses: implications for global measles elimination programs. Emerg Infect Dis. 1998;4:29–35. 9452396 10.3201/eid0401.980105 PMC2627654

[10] Rota JR, Bellini WJ, Rota PA. Measles. In: Thompson RCA, editor. Molecular epidemiology of infectious diseases. London: Kluwer Academic & Lippincott Raven Publishers; 2000. p. 168–80.

[11] World Health Organization. Standardization of the nomenclature for describing the genetic characteristics of wild-type measles viruses. Wkly Epidemiol Rec. 1998;73:265–72. 9745371

[12] World Health Organization. Nomenclature for describing the genetic characteristics of wild-type measles viruses (update). Part I. Wkly Epidemiol Rec. 2001;76:242–7. 11515240

[13] World Health Organization. Nomenclature for describing the genetic characteristics of wild-type measles viruses (update). Wkly Epidemiol Rec. 2001;76:249–51. 11561558

[14] Baseler MW, Stevens RA, Metcalf JA. Immunologic monitoring of patients with human immunodeficiency virus. In: Rose NR, DeMarcario EC, Fahay JL, Friedman H, Penn GM, editors. Manual of clinical laboratory immunology. 4th ed. Washington: American Society for Microbiology; 1992. p. 377.

[15] Kobune F, Sakata H, Sugiura A. Marmoset lymphoblastoid cells as a sensitive host for isolation of measles virus. J Virol. 1990;64:700–5. 2153236 10.1128/jvi.64.2.700-705.1990 PMC249163

[16] Chomczynski P, Sacchi N. Single-step method of RNA isolation by acid guanidinium thiocyanate-phenol-chloroform extraction. Anal Biochem. 1987;162:156–9. 10.1016/0003-2697(87)90021-2 2440339

[17] Liffick S, Thoung N, Xu W, Li Y, Lien H, Bellini WJ, Genetic characterization of contemporary wild-type viruses from Vietnam and China: identification of two genotypes within clade H. Virus Res. 2001;77:81–7. 10.1016/S0168-1702(01)00268-4 11451490

[18] Devereux J, Haeberli P, Smithies O. A comprehensive set of sequence analysis programs for the VAX. Nucleic Acids Res. 1984;12:387–95. 10.1093/nar/12.1Part1.387 6546423 PMC321012

[19] Swofford DL. PAUP: phylogenetic analysis using parsimony. Version 3.1.1 Champaign (IL): Illinois Natural History Survey; 1986.

[20] Oliveira I, Curti SP, Figueiredo CA, Afonso MAS, Theobalso M, Souza LTM, Genetic analysis of measles virus in São Paulo, Brazil. Virus Rev Res. 1998;3:7–8.

[21] Siqueira MM, Castro-Silva R, Cruz C, Oliveira IC, Cunha GMC, Mello M, Genomic characterization of wild-type measles viruses that circulated in different states during the 1997 measles epidemic. J Med Virol. 2001;63:299–304. 10.1002/1096-9071(200104)63:4<299::AID-JMV1005>3.0.CO;2-3 11241461

[22] Barrero PR, de Wolff CD, Passeggi CA, Mistchenko AS. Sequence analysis of measles virus hemagglutinin isolated in Argentina during the 1997–1998 outbreak. J Med Virol. 2000;60:91–6. 10.1002/(SICI)1096-9071(200001)60:1<91::AID-JMV15>3.0.CO;2-M 10568769

[23] Baumeister E, Siqueira MM, Savy V, Friedrich F. Genetic characterization of wild-type measles viruses isolated during the 1998 measles epidemic in Argentina. Acta Virol. 2000;44:169–74. 11155360

[24] Canepa E, Siqueira M, Hortal M, Friedrich F. Recent measles viral activity in Uruguay: serological and genetic approaches. Acta Virol. 2000;44:35–9. 10989690

[25] Rota PA, Liffick SL, Rota JS, Katz RS, Redd S, Papania M, Molecular epidemiology of measles viruses in the United States: 1997–2001. Emerg Infect Dis. 2002. In press. 10.3201/eid0809.020206 PMC2732556 12194764

[26] Rota JS, Rota PA, Redd SB, Pattamadilok S, Bellini WJ. Genetic analysis of measles viruses isolated in the United States, 1995-1996. J Infect Dis. 1998;177:204–8. 10.1086/513825 9419189

[27] Santibanez S, Heider A, Gerike E, Agafonov A, Schreier E. Genotyping of measles virus isolates from central Europe and Russia. J Med Virol. 1999;58:313–20. 10.1002/(SICI)1096-9071(199907)58:3<313::AID-JMV19>3.0.CO;2-P 10447429

[28] Hanses F, van Binnendijk R, Ammerlaan W, Truong AT, de Rond L, Schneider F, Genetic variability of measles viruses circulating in the Benelux. Arch Virol. 2001;145:541–51. 10.1007/s007050050045 10795521

[29] Rima BK, Earle JAP, Yeo RP, Herlihy L, Baczko K, ter Meulen V, Temporal and geographical distribution of measles virus genotypes. J Gen Virol. 1995;76:1173–80. 10.1099/0022-1317-76-5-1173 7730801

[30] Rima BK, Earle JAP, Baczko K, ter Meulen V, Liebert U, Carstens C, Sequence divergence of measles virus hemagglutinin during natural evolution and adaptation to cell culture. J Gen Virol. 1997;78:97–106. 9010291 10.1099/0022-1317-78-1-97

[31] Jin L, Brown DWG, Ramsay MEB, Rota PA, Bellini WJ. The diversity of measles virus in the UK, 1992–1995. J Gen Virol. 1997;78:1287–94. 9191920 10.1099/0022-1317-78-6-1287

[32] Yamaguchi S. Identification of three lineages of wild measles virus by nucleotide sequence analysis of the N, P, M, F and L genes in Japan. J Med Virol. 1997;52:113–26. 10.1002/(SICI)1096-9071(199705)52:1<113::AID-JMV18>3.0.CO;2-N 9131467

[33] Katayama Y, Shibahara K, Kohama T, Homma M, Hotta H. Molecular epidemiology and changing distribution of genotypes of measles virus field strains in Japan. J Clin Microbiol. 1997;35:2651–63. 9316925 10.1128/jcm.35.10.2651-2653.1997 PMC230028

[34] Redd SC, Markowitz LE, Katz SL. In: Plotkin SA, Orenstein WA, editors. Vaccines 3rd ed. Philadelphia: WB Saunders & Co.; 1999. p. 222–66.

[35] Centers for Disease Control and Prevention. Absence of reported measles—United States. MMWR Morb Mortal Wkly Rep. 1993;48:925–6. 8246853

[36] Centers for Disease Control and Prevention. Progress toward interrupting indigenous measles transmission: Region of the Americas, January 1999. MMWR Morb Mortal Wkly Rep. 2000;49:986–90. 11098862

[37] Centers for Disease Control and Prevention. Measles—United States, 1996, and the interruption of indigenous transmission. MMWR Morb Mortal Wkly Rep. 1997;46:242–6. 9082179

[38] Pan American Health Organization. Measles in El Salvador. EPI Newsl. 2001;23:1–3.

